# SGLT2i impact on HCC incidence in patients with fatty liver disease and diabetes: a nation-wide cohort study in South Korea

**DOI:** 10.1038/s41598-024-60133-3

**Published:** 2024-04-29

**Authors:** Hyo Jung Cho, Eunyoung Lee, Soon Sun Kim, Jae Youn Cheong

**Affiliations:** 1 Department of Gastroenterology, Ajou University School of Medicine, Worldcup-ro 164, Yeongtong-gu, Suwon, 16499 Republic of Korea; 2 Department of Neurology, McGovern Medical School at UTHealth, Houston, TX USA

**Keywords:** Hepatocellular carcinoma, Sodium-glucose cotransporter-2 inhibitors (SGLT2i), Fatty liver, Type 2 diabetes mellitus, Chronic viral hepatitis, Cancer, Drug discovery, Endocrinology, Gastroenterology

## Abstract

This study evaluated the effect of sodium-glucose cotransporter-2 inhibitors (SGLT2i) on cancer development, particularly in hepatocellular carcinoma (HCC), in individuals with concomitant fatty liver disease (FLD) and type 2 diabetes mellitus (T2DM). Using data from Korea's Health Insurance Review and Assessment Service, we performed Kaplan–Meier and Cox regression analyses in patients with non-alcoholic fatty liver disease (NAFLD) and T2DM (NAFLD-T2DM cohort) and those with chronic viral hepatitis (CVH) alongside FLD and T2DM (FLD-T2DM-CVH cohort). In the propensity score (PS) matched NAFLD-T2DM cohort (N = 107,972), SGLT2i use was not associated with the occurrence of overall cancer, including HCC. However, old age, male sex, liver cirrhosis, and hypothyroidism were identified as independent risk factors for HCC occurrence, whereas statin and fibrate usage were associated with reduced HCC risk in this cohort in multivariate Cox analysis. In the PS-matched FLD-T2DM-CVH cohort (N = 2798), a significant decrease in HCC occurrence was observed among SGLT2i users (*P* = 0.03). This finding remained consistent in the multivariate Cox regression analysis (Hazard ratio = 2.21, 95% confidence interval = 1.01–4.85, *P* = 0.048). In conclusion, SGLT2i may be a beneficial option for diabetes management in patients with concomitant T2DM, FLD, and CVH while affirming the overall safety of SGLT2i in other types of cancer.

## Introduction

Fatty liver disease (FLD), which is characterized by the accumulation of fat in the liver, is a prevalent liver disorder with significant global impact^1,2^. This condition is a hepatic manifestation of metabolic syndrome and is closely linked to insulin resistance and type 2 diabetes mellitus (T2DM)^2–4^. Individuals with diabetes are at a higher risk of developing FLD, and diabetes increases the risk of progression to more severe liver diseases, such as liver cirrhosis or hepatocellular carcinoma (HCC)^5,6^. Various studies have indicated that patients with concurrent FLD and T2DM are significantly more likely to develop HCC^6–8^. With the incidence of FLD and T2DM increasing worldwide, managing the risk of progression to HCC in these patient populations is becoming a critical concern.

Sodium-glucose cotransporter-2 inhibitors (SGLT2i) are a class of drugs primarily used to treat T2DM^9^. Beyond their role in lowering blood glucose levels, emerging research suggests SGLT2i may offer additional benefits in liver diseases, including non-alcoholic fatty liver disease (NAFLD) and HCC^10–12^. Pre-clinical studies have shown that SGLT2i can decrease hepatic steatosis, enhance insulin sensitivity, and reduce liver inflammation and fibrosis^13,14^. Several clinical studies have shown that the use of SGLT2i in patients with NAFLD improves liver function and serum markers of liver injury^11,15^. Additionally, the use of SGLT2i in T2DM patients has been observed to reduce the risk of HCC development^16^. However, the relationship between SGLT2i and other cancer types has yielded mixed outcomes; while some studies report a reduced risk of cancer, such as lung and gastrointestinal cancers, others have raised concerns over increased risks of bladder cancer^17^. Given the relatively recent introduction of SGLT2i in the market, there is a critical need for further research involving long-term follow-up and the use of clinical big data to more thoroughly investigate the cancer incidence associated with SGLT2i use.

In healthcare research, the use of big data has become increasingly vital, particularly for identifying trends, patterns, and correlations within vast datasets^18^. Our study used data from the Health Insurance Review and Assessment Service (HIRA) of Korea, which is an extensive database encompassing a wide array of patient information^19^. The use of such big datasets offers a unique opportunity to conduct comprehensive and detailed analyses of large populations. This approach enabled us to observe real-world outcomes, overcome the limitations of small sample sizes, and enhance the generalizability of our findings.

Our study aimed to evaluate the impact of SGLT2i on cancer development, with a specific focus on HCC, in patients with co-existing FLD and T2DM, using a nationwide Korean cohort from the HIRA. We conducted our analysis on two distinct subpopulations of patients coexisting with FLD and T2DM. The first cohort included patients with T2DM and NAFLD, after excluding those with other chronic liver diseases, such as chronic viral hepatitis (CVH), alcoholic liver disease, and autoimmune liver disease from patients with FLD and T2DM. The second cohort comprised high-risk individuals with HCC who were diagnosed with CVH among patients with FLD and T2DM. In addition to assessing the impact of SGLT2i on HCC incidence, we also examined various demographic and clinical factors to identify independent risk factors for HCC in these patient groups, leveraging an extensive dataset to provide insights into effective HCC risk management strategies.

## Results

### Baseline characteristics and incidence rate of cancers in the NAFLD-T2DM cohort

**Figure 1:**
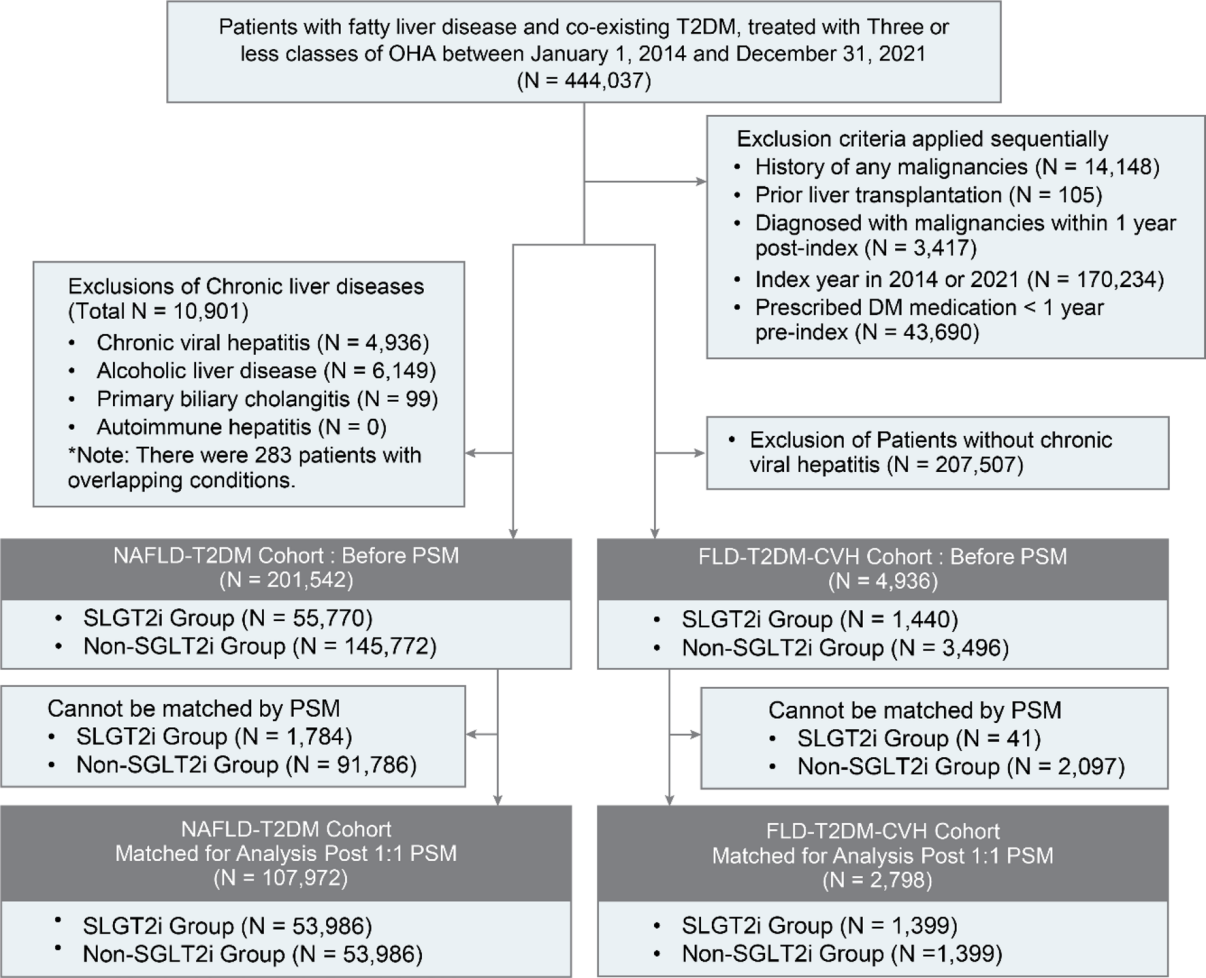
Schematic representation of cohort derivation for this study. *T2DM* type 2 diabetes mellitus, *NAFLD* non-alcoholic fatty liver disease, *FLD* fatty liver disease, *CVH* chronic viral hepatitis, *SGLT2i* sodium-glucose cotransporter-2 inhibitor, *PSM* propensity score matching.

We identified 201,542 patients with co-existing NAFLD and T2DM. Of these patients, 55,770 (27.7%) were in the SGLT2i group and 145,772 (72.3%) were in the non-SGLT2i group. The median [interquartile range (IQR)] of the follow-up time was 3.56 (2.17–5.10) years for all, 3.01 (1.94–4.52) years for SLGT2i group, and 3.77 (2.30–5.34) years for Non-SGLT2i group. This selection was conducted after excluding patients diagnosed with chronic liver diseases including CVH, alcoholic liver disease, and autoimmune liver disease. After 1:1 PS matching, a balanced cohort of 107,972 patients was established for analysis and evenly divided into 53,986 patients (50.0%) in the SGLT2i group and 53,986 patients (50.0%) in the non-SGLT2i group (Fig. 1). In PS-matched cohort, the median (IQR) of the follow-up time was 3.04 (1.94–4.55) years for all, 3.05 (1.95–4.56) years for SLGT2i group, and 3.03 (1.93–4.54) years for Non-SGLT2i group. There was no significant difference in follow-up period between the two groups (*P* = 0.445).

Supplementary Table 1 and Figure S1A (Love plot) confirm the successful adjustment of covariate differences between groups following PS matching. In this cohort, PS matching effectively standardized the mean differences, with all variables achieving an aSMD of less than 0.1, demonstrating excellent balance across covariates. These results underscore the robustness of the matching process and comparability of the groups for subsequent analyses. Table 1 illustrates comprehensive patient characteristics before and after PS matching.

**Table 1:** Baseline characteristics of pre- and post-PS matched NAFLD-T2DM cohort.

Characteristics	Pre-PS matching cohort			Post-PS matched cohort		
	SGLT2i users (N = 55,770)	Non-SGLT2i users (N = 145,772 )	aSMD	SGLT2i users (N = 53,986 )	Non-SGLT2i users (N = 53,986)	aSMD
Age, years (mean ± SD)	49.72 (12.91)	56.29 (12.02)	0.527	50.37 (12.55)	50.61 (12.48)	0.02
Sex, male, n (%)	31,703 (56.8)	84,868 (58.2)	0.028	30,697 (56.9)	30,731 (56.9)	0.001
Index year						
2015	4160 (7.5)	22,956 (15.7)	0.354	4153 (7.7)	3977 (7.4)	0.013
2016	6697 (12.0)	25,095 (17.2)		6649 (12.3)	6625 (12.3)	
2017	8890 (15.9)	24,021 (16.5)		8699 (16.1)	8747 (16.2)	
2018	9716 (17.4)	24,204 (16.6)		9463 (17.5)	9438 (17.5)	
2019	13,071 (23.4)	24,555 (16.8)		12,420 (23.0)	12,467 (23.1)	
2020	13,236 (23.7)	24,941 (17.1)		12,602 (23.3)	12,732 (23.6)	
CCI index						
0	3631 (6.5)	13,236 (9.1)	0.108	3613 (6.7)	3602 (6.7)	0.005
1	9376 (16.8)	26,568 (18.2)		9151 (17.0)	9187 (17.0)	
2	16,349 (29.3)	40,424 (27.7)		15,755 (29.2)	15,856 (29.4)	
≤ 3	26,414 (47.4)	65,544 (45.0)		25,467 (47.2)	25,341 (46.9)	
Comorbidities						
Hypertension	25,196 (45.2)	66,474 (45.6)	0.008	24,703 (45.8)	22,681 (42.0)	0.076
Dyslipidemia	35,496 (63.6)	83,122 (57.0)	0.136	34,180 (63.3)	34,161 (63.3)	0.001
Cerebrovascular disease	1580 (2.8)	3175 (2.2)	0.042	1557 (2.9)	938 (1.7)	0.076
Coronary artery disease	1281 (2.3)	4561 (3.1)	0.051	1272 (2.4)	1351 (2.5)	0.01
Peripheral vascular disease	1642 (2.9)	3140 (2.2)	0.05	1633 (3.0)	859 (1.6)	0.096
Heart failure	4073 (7.3)	13,074 (9.0)	0.061	4021 (7.4)	4390 (8.1)	0.026
Hypothyroidism	2202 (3.9)	5030 (3.5)	0.026	2121 (3.9)	1977 (3.7)	0.014
Obesity or overweight	262 (0.5)	214 (0.1)	0.058	230 (0.4)	121 (0.2)	0.035
Chronic kidney disease	352 (0.6)	879 (0.6)	0.004	331 (0.6)	320 (0.6)	0.003
Liver cirrhosis	131 (0.2)	405 (0.3)	0.008	129 (0.2)	140 (0.3)	0.004
Chronic respiratory disease	351 (0.6)	878 (0.6)	0.003	330 (0.6)	320 (0.6)	0.002
Co-medications						
Aspirin	6526 (11.7)	18,618 (12.8)	0.033	6492 (12.0)	5193 (9.6)	0.078
Clopidogrel	2698 (4.8)	6312 (4.3)	0.024	2685 (5.0)	1818 (3.4)	0.08
Antiplatelet, others	1880 (3.4)	5933 (4.1)	0.037	1872 (3.5)	1854 (3.4)	0.002
Statin	24,068 (43.2)	57,996 (39.8)	0.068	23,579 (43.7)	22,249 (41.2)	0.05
Ezetimibe	5353 (9.6)	9952 (6.8)	0.101	5217 (9.7)	4573 (8.5)	0.042
Fibrates	3729 (6.7)	8063 (5.5)	0.048	3600 (6.7)	3875 (7.2)	0.02
ACEi	632 (1.1)	1327 (0.9)	0.022	622 (1.2)	355 (0.7)	0.052
ARB	18,006 (32.3)	46,487 (31.9)	0.008	17,701 (32.8)	16,357 (30.3)	0.054
CCB	11,793 (21.1)	33,246 (22.8)	0.04	11,591 (21.5)	11,362 (21.0)	0.01
Beta blocker	5628 (10.1)	13,738 (9.4)	0.022	5500 (10.2)	4576 (8.5)	0.059
Level of T2DM treatment						
Level I	47,658 (85.5)	127,376 (87.4)	0.066	46,278 (85.7)	46,332 (85.8)	0.005
Level II	3203 (5.7)	8108 (5.6)		3096 (5.7)	3121 (5.8)	
Level III	4909 (8.8)	10,288 (7.1)		4612 (8.5)	4533 (8.4)	

*SGLT2i* sodium-glucose cotransporter-2 inhibitor, *NAFLD* non-alcoholic fatty liver disease, *T2DM* type 2 diabetes mellitus, *CVH* chronic viral hepatitis, *PSM* propensity score, *aSMD* absolute standardized mean difference, *CCI* Charlson Comorbidity Index, *ACEi* angiotensin-converting enzyme inhibitor, *ARB* angiotensin II receptor blocker, *CCB* calcium channel blocker.

Table 2 displays the number of cancer cases, person-years, and IR per 10,000 person-years [95% CI] for each type of cancer according to SGLT2i exposure status in the NAFLD-T2DM cohort both before and after PS matching. Figure 3A shows a forest plot of the HRs for each cancer type. In the pre-matching analysis, non-SGLT2i users exhibited significant HRs for the occurrence of “total cancer”, HCC, CCC, stomach cancer, colorectal cancer, pancreatic cancer, lung cancer, prostate cancer, and “other cancers”. However, after PS matching, the statistically significant differences in cancer risk between the two groups disappeared.

**Table 2:** Incident rate per 10,000 person year of the malignancies according to SGLT2i usage in the pre- and post-PS matched NAFLD-T2DM cohort.

	Pre-PS matched cohort (N = 201,542)						Post-PS matched cohort (N = 107,972)					
	Cancer cases		Person-year		Incidence rate per 10,000 person year (95% CI)		Cancer cases		Person-year		Incidence rate per 10,000 person year (95% CI)	
	SGLT2i users	Non-SGLT2i users	SGLT2i users	Non-SGLT2i users	SGLT2i users	Non-SGLT2i users	SGLT2i users	Non-SGLT2i users	SGLT2i users	Non-SGLT2i users	SGLT2i users	Non-SGLT2i users
Total cancer	1046	4535	186,705.42	566,243.17	56.0 (52.7–59.5)	80.1 (77.8–82.5)	1036	1034	182,187.4	181,929.2	56.9 (53.5–60.4)	56.8 (53.5–60.4)
Hepatocellular carcinoma	67	326	188,137.25	573,129.17	3.6 (2.8–4.5)	5.7 (5.1–6.3)	67	59	183,610.3	183,367.3	3.6 (2.9–4.6)	3.2 (2.4–4.2)
Cholangiocarcinoma	4	50	188,235.58	573,559.92	0.2 (0.1–0.6)	0.9 (0.7–1.2)	4	6	183,708.7	183,435.2	0.2 (0.1–0.6)	0.3.(0.2–0.7)
Stomach cancer	97	567	188,094.25	572,564.67	5.2 (4.2–6.3)	9.9 (9.1–10.8)	97	124	183,567.3	183,245.5	5.3 (4.3–6.4)	6.8 (5.7–8.1)
Colorectal cancer	101	489	188,081.25	572,736.75	5.4 (4.4–6.5)	8.5 (7.8–9.3)	99	109	183,556.6	183,270.1	5.4 (4.4–6.6)	5.9 (4.9–7.2)
Esophagus cancer	6	44	188,228.5	573,546.83	0.3 (0.1–0.7)	0.8 (0.6–1.0)	6	6	183,701.6	183,432.8	0.3 (0.1–0.7)	0.3 (0.1–0.7)
Pancreas cancer	37	196	188,205	573,411.92	2.0 (1.4–2.7)	3.4 (3.0–3.9)	37	41	183,678.1	183,393.4	2.0 (1.5–2.8)	2.2 (1.6–3.0)
Lung cancer	89	413	188,127.83	573,053.08	4.7 (3.8–5.38)	7.2 (6.5–7.9)	89	66	183,600.9	183,353.5	4.8 (3.9–6.0)	3.6 (2.8–4.6)
Bladder cancer	43	149	188,177.67	573,304.5	2.3 (1.7–3.1)	2.6 (2.2–3.1)	43	42	183,650.8	183,371.4	2.3 (1.7–3.2)	5.7 (2.3–1.7)
Prostate cancer	52	329	103,664.42	332,421.75	5.0 (3.8–6.6)	9.9 (8.9–11.0)	52	44	101,202.6	103,408.4	5.1 (3.9–6.7)	4.3 (3.2–5.7)
Breast cancer	118	337	84,300.58	240,001.5	14.0 (11.7–16.8)	14.0 (12.6–15.6)	118	118	82,235.5	79,776	14.4 (120–17.2)	14.8 (12.3–17.7)
Cervical cancer	10	45	84,477.75	240,560.92	1.2 (0.6–2.2)	1.9 (0.4–2.5)	9	9	82,416.08	79,955.67	1.1 (0.6–2.1)	1.1 (0.6–2.2)
Other cancers	422	1590	187,603.42	571,036.08	22.5 (20.4–24.7)	27.8 (26.5–29.2	415	410	183,079.8	182,834.8	22.7 (20.6–25.0)	22.4 (20.4–24.7)

*SGLT2i* sodium-glucose cotransporter-2 inhibitor, *NAFLD* non-alcoholic fatty liver disease, *T2DM* type 2 diabetes mellitus, *CVH* chronic viral hepatitis, *PSM* propensity score matching, *HR* hazard ratio, *CI* confidence interval.

### Survival analysis of HCC and other cancers in the PS-matched NAFLD-T2DM cohort according to SGLT2i usage

**Figure 2:**
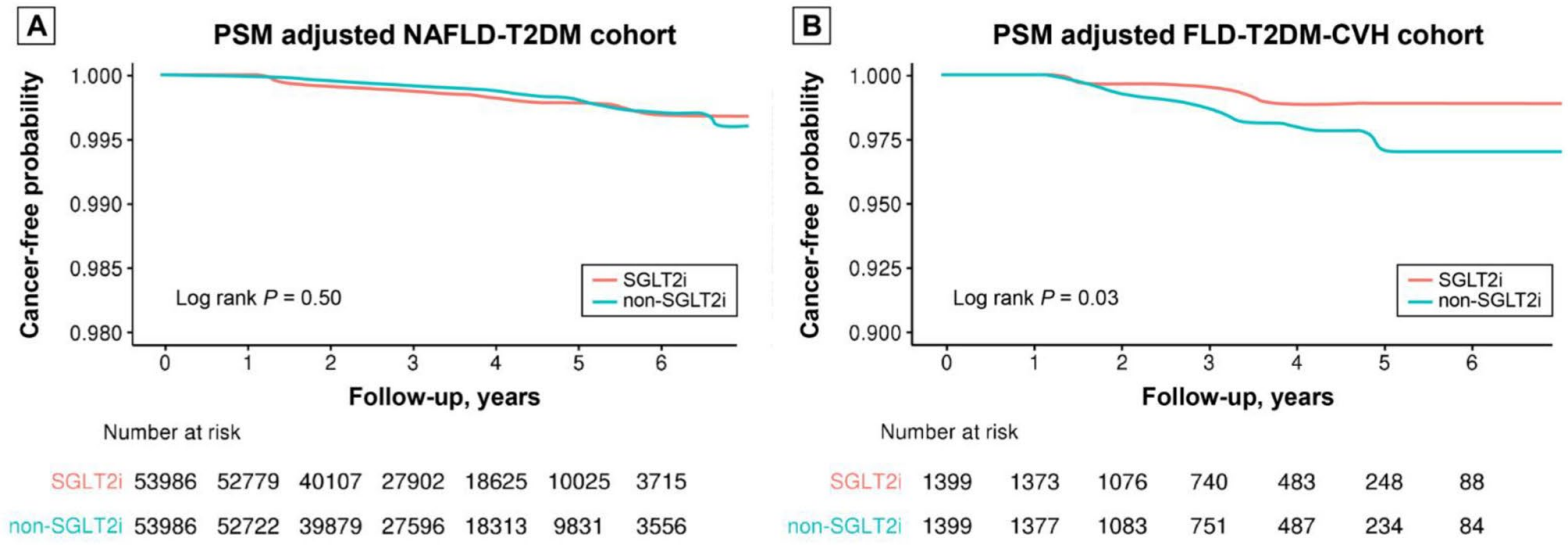
Comparison of Kaplan–Meier curves of HCC occurrence according to SGLT2i exposure in PS-matched NAFLD-T2DM cohort and PS-matched FLD-T2DM-CVH cohort. *HCC* hepatocellular carcinoma, *SGLT2i* sodium-glucose cotransporter-2 inhibitor, *PS* propensity score, *NAFLD* non-alcoholic fatty liver disease, *T2DM* type 2 diabetes mellitus, *FLD* fatty liver disease, *CVH* chronic viral hepatitis.

Figure 2A shows the Kaplan–Meier curves comparing the probability of HCC incidence between SGLT2i users and non-SGLT2i users within the PS-matched NAFLD-T2DM cohort. No significant differences were observed not only in HCC development but also in other types of cancer, indicating that SGLT2i usage does not statistically influence cancer incidence in this cohort (Fig. S1).

Subsequently, Cox proportional hazards analysis was conducted to identify the independent variables affecting HCC occurrence in this cohort (Table 3). In the univariate Cox regression analysis, older age; male sex; comorbidities such as hypertension, hypothyroidism, and liver cirrhosis; and the use of aspirin, beta-blockers, calcium channel blockers, and fibrates were identified as significant risk factors for HCC occurrence. In the multivariate Cox regression analysis, which included variables with a *P* value < 0.1 and SGLT2i used from the univariate analysis, older age (HR = 1.08, 95% CI = 1.06–1.10, *P* < 0.001), male sex (HR = 2.79, 95% CI = 1.87–4.14, *P* < 0.001), hypothyroidism (HR = 2.43, 95% CI = 1.21–4.87, *P* = 0.013), liver cirrhosis (HR = 17.88, 95% CI = 8.19–39.03, *P* < 0.001), statin use (HR = 0.59, 95% CI = 0.36–0.96, *P* = 0.035), and fibrate use (HR = 0.14, 95% CI = 0.02–0.99, *P* = 0.049) were identified as independent risk factors for HCC occurrence. The Concordance index of this model was 0.805, with a standard error (SE) of 0.029.

**Table 3:** Univariate and multivariate Cox regression analysis to identify risk factors associated with HCC occurrence in the PS-matched NAFLD-T2DM cohort.

Variables	Univariate			Multivariate		
	HR	95% CI	P value	HR	95% CI	P value
Age, year	1.07	1.05–1.09	< 0.001	1.08	1.06–1.10	< 0.001
Sex, female	1.97	1.34–2.90	< 0.001	2.79	1.87–4.14	< 0.001
SGLT2i use, no	0.88	0.62–1.45	0.472	0.87	0.61–1.24	0.435
CCI index, 0 or 1 vs. ≥ 2	1.20	0.77–1.85	0.424			
Comorbidities						
Hypertension	1.70	1.20–2.42	0.003	1.30	0.75–2.26	0.349
Dyslipidemia	1.37	0.51–1.04	0.079	0.71	0.45–1.12	0.138
Cerebrovascular disease	0.80	0.20–3.24	0.756			
Peripheral vascular disease	1.00	0.32–3.16	0.994			
Heart failure	0.94	0.48–1.86	0.868			
Hypothyroidism	2.00	1.02–3.94	0.045	2.43	1.21–4.87	0.013
Chronic kidney disease	1.58	0.22–11.28	0.650			
Chronic respiratory disease	2.06	0.51–8.31	0.312			
End stage renal disease	1.58	0.22–11.30	0.649			
Liver cirrhosis	29.71	13.86–63.70	< 0.001	17.88	8.19–39.03	< 0.001
Co-medications						
Aspirin	1.60	1.01–2.54	0.044	1.01	0.61–1.67	0.972
Clopidogrel	1.72	0.84–3.52	0.138			
Statin	0.71	0.50–1.04	0.080	0.59	0.36–0.96	0.035
Ezetimibe	0.35	0.11–1.11	0.074	0.44	0.14–1.42	0.170
Fibrates	0.13	0.02–0.93	0.043	0.14	0.02–0.99	0.049
Angiotensin-converting enzyme inhibitor	0.80	0.11–5.71	0.822			
Angiotensin II receptor blocker	1.39	0.97–2.00	0.071	0.95	0.57–1.57	0.838
Calcium channel blocker	1.59	1.08–2.35	0.018	1.08	0.67–1.74	0.743
Beta-blocker	1.70	1.02–2.77	0.041	1.13	0.67–1.92	0.643
Metformin	1.22	0.80–1.86	0.350			
Level of antidiabetic treatment, level 1 vs. ≥ 2	1.31	0.81–2.11	0.276			

*PS* propensity score, *NAFLD* non-alcoholic fatty liver disease, *T2DM* type 2 diabetes mellitus, *HR* hazard ratio, *CI* confidence interval, *SGLT2i* sodium-glucose cotransporter-2 inhibitor, *CCI* Charlson Comorbidity Index.

Concordance = 0.806 (standard error = 0.029).

### Baseline characteristics and incidence rate of cancers in the FLD-T2DM-CVH cohort

In this subset, 4936 patients with CVH along with co-existing NAFLD and T2DM were identified. Among them, 1440 (29.2%) were categorized into the SGLT2i group and 3,496 (70.8%) into the non-SGLT2i group. The median (IQR) of the follow-up period was 3.50 (2.18–4.95) years for all, 3.06 (2.04–4.46) years for SLGT2i group, and 3.67 (2.25–5.17) years for Non-SGLT2i group. Following 1:1 PS matching, an eligible cohort for analysis was formed, consisting of patients with an equal distribution of 1,399 patients (50.0%) in both the SGLT2i and non-SGLT2i groups (Fig. 1). In the PS-matched FLD-T2DM-CVH cohort, the median [IQR] of the follow-up time was 3.13 (2.07–4.48) years for all, 3.07 (2.03–4.48) years for SLGT2i group, and 3.19 (2.10–4.48) years for non-SGLT2i group. There was no significant difference in follow-up period between the two groups (*P* = 0.529). 

Supplementary Table 2 and Fig. S1B (Love plot) confirm the successful adjustment of covariate differences between groups following PS matching. Significant discrepancies between groups were noted before PS matching; however, PS matching effectively standardized the mean differences in the PS-matched cohort, with all variables achieving an aSMD of less than 0.1, demonstrating excellent balance across covariates. These results underscore the robustness of the matching process and comparability of the groups for subsequent analyses. Table 4 illustrates comprehensive patient characteristics before and after PS matching.

**Table 4:** Baseline characteristics of pre- and post-PS matched FLD-T2DM-CVH cohort.

characteristics	Pre-PS matched cohort (N = 4936)			Post-PS matched cohort (N = 2798)		
	SGLT2i users (N = 1440)	Non-SGLT2i users (N = 3.496 )	aSMD	SGLT2i users (N = 1399 )	Non-SGLT2i users (N = 1399)	aSMD
Age, years (mean ± SD)	51.76 (11.19)	56.45 (10.82)	0.427	52.27 (10.85)	52.31 (10.99)	0.004
Sex, male, n (%)	848 (58.9)	2197 (62.8)	0.081	831 (59.4)	828 (59.2)	0.004
Index year						
2015	99 (6.9)	535 (15.3)	0.341	98 (7.0)	101 (7.2)	0.042
2016	172 (11.9)	577 (16.5)		172 (12.3)	164 (11.7)	
2017	251 (17.4)	577 (16.5)		243 (17.4)	258 (18.4)	
2018	259 (18.0)	582 (16.6)		251 (17.9)	259 (18.5)	
2019	363 (25.2)	609 (17.4)		345 (24.7)	328 (23.4)	
2020	296 (20.6)	616 (17.6)		290 (20.7)	289 (20.7)	
CCI						
0	0 (0)	0 (0)	0.083	0 (0)	0 (0)	0.02
1	49 (3.4)	161 (4.6)		48 (3.4)	50 (3.6)	
2	397 (27.6)	1039 (29.7)		392 (28.0)	380 (27.2)	
≤ 3	994 (69.0)	2296 (65.7)		959 (68.5)	969 (69.3)	
Comorbidities						
Hypertension	779 (54.1)	1838 (52.6)	0.031	758 (54.2)	759 (54.3)	0.001
Dyslipidemia	1143 (79.4)	2454 (70.2)	0.213	1104 (78.9)	1106 (79.1)	0.004
Heart failure	64 (4.4)	108 (3.1)	0.071	60 (4.3)	58 (4.1)	0.007
Cerebrovascular disease	51 (3.5)	150 (4.3)	0.039	50 (3.6)	62 (4.4)	0.044
Coronary artery disease	64 (4.4)	98 (2.8)	0.088	62 (4.4)	47 (3.4)	0.055
Peripheral vascular disease	138 (9.6)	407 (11.6)	0.067	132 (9.4)	170 (12.2)	0.088
Hypothyroidism	116 (8.1)	231 (6.6)	0.056	112 (8.0)	101 (7.2)	0.03
Obesity or overweight	5 (0.3)	10 (0.3)	0.011	4 (0.3)	5 (0.4)	0.013
Chronic kidney disease	17 (1.2)	35 (1.0)	0.017	17 (1.2)	19 (1.4)	0.013
Alcoholic liver disease	65 (4.5)	205 (5.9)	0.061	63 (4.5)	56 (4.0)	0.025
Primary biliary cholangitis	2 (0.1)	2 (0.1)	0.026	1 (0.1)	1 (0.1)	< 0.001
Chronic viral hepatitis B	1018 (70.7)	2386 (68.2)	0.053	992 (70.9)	991 (70.8)	0.002
Chronic viral hepatitis C	196 (13.6)	638 (18.2)	0.127	192 (13.7)	187 (13.4)	0.01
Liver cirrhosis	24 (1.7)	64 (1.8)	0.013	66 (4.7)	63 (4.5)	0.01
Co-medications						
Aspirin	169 (11.7)	450 (12.9)	0.035	167 (11.9)	154 (11.0)	0.029
Clopidogrel	76 (5.3)	165 (4.7)	0.026	75 (5.4)	71 (5.1)	0.013
Antiplatelet, others	72 (5.0)	148 (4.2)	0.037	69 (4.9)	58 (4.1)	0.038
Statin	681 (47.3)	1442 (41.2)	0.122	665 (47.5)	655 (46.8)	0.014
Ezetimibe	162 (11.2)	259 (7.4)	0.132	156 (11.2)	156 (11.2)	< 0.001
Fibrates	97 (6.7)	187 (5.3)	0.058	93 (6.6)	84 (6.0)	0.026
ACEi	28 (1.9)	35 (1.0)	0.078	25 (1.8)	23 (1.6)	0.011
ARB	579 (40.2)	1291 (36.9)	0.067	565 (40.4)	574 (41.0)	0.013
CCB	364 (25.3)	918 (26.3)	0.022	355 (25.4)	377 (26.9)	0.036
Beta blocker	182 (12.6)	405 (11.6)	0.032	176 (12.6)	170 (12.2)	0.013
Level of T2DM treatment						
Level I	1212 (84.2)	3080 (88.1)	0.121	1182 (84.5)	1193 (85.3)	0.04
Level II	74 (5.1)	157 (4.5)		70 (5.0)	75 (5.4)	
Level III	154 (10.7)	259 (7.4)		147 (10.5)	131 (9.4)	

*PS* propensity score, *FLD* fatty liver disease, *T2DM* type 2 diabetes mellitus, *CVH* chronic viral hepatitis, *SGLT2i* sodium-glucose cotransporter-2 inhibitor, *aSMD* absolute standardized mean difference, *CCI* Charlson Comorbidity Index, *ACEi* angiotensin-converting enzyme inhibitor, *ARB* angiotensin II receptor blocker, *CCB* calcium channel blocker.

Table 5 shows the number of cancer cases, person-years, and IR per 10,000 person-years for each cancer type according to SGLT2i exposure in the FLD-T2DM-CVH cohort before and after PS matching. Notably, the crude IR per 10,000 person-years of HCC was significantly higher in the FLD-T2DM-CVH cohort (IR per 10,000 person-years: 57.3, 95% CI 18.4–71.6) compared to the NAFLD-T2DM cohort (IR per 10,000 person-years: 5.2, 95% CI 3.6–5.7). Interestingly, in both pre-and post-PS matching, the IR per 10,000 person-years of HCC was markedly higher in the non-SGLT2i group (Pre-PS matching: 18.4 vs 71.6, and post-PS matching: 18.8 vs 41.7, for SGLT2i users and non-SGLT2i users, respectively).

**Table 5:** Incident rate per 10,000 person year of the malignancies according to SGLT2i use in the pre- and post-PS matched FLD-T2DM-CVH cohort.

	Pre-PS matched cohort (N = 4936)						Post-PS matched cohort (N = 2798)					
	Cancer cases		Person-year		Incidence rate per 10,000 person year (95% CI)		Cancer cases		Person-year		Incidence rate per 10,000 person year (95% CI)	
	SGLT2i users	Non-SGLT2i users	SGLT2i users	Non-SGLT2i users	SGLT2i users	Non-SGLT2i users	SGLT2i users	Non-SGLT2i users	SGLT2i users	Non-SGLT2i users	SGLT2i users	Non-SGLT2i users
Total cancers	38	194	4850.3	13,238.8	78.3 (57.0–107.7)	146.5 (127.3–168.7)	38	37	4727.3	4772.8	80.4 (58.5–110.5)	77.5 (56.2–107.0)
Hepatocellular carcinoma	9	96	4900.2	13,412.1	18.4 (9.6–35.3)	71.6 (58.6–87.4)	9	21	4777.3	4804.1	18.8 (9.8–36.2)	41.7 (28.5–67.0)
Cholangiocarcinoma	1	3	4908.4	13,557.8	2.0 (0.3–14.5)	2.2 (0.7–6.9)	1	1	4785.5	4839.1	2.1 (0.3–14.8)	2.1 (0.3–14.7)
Stomach cancer	4	11	4905	13,543.1	8.2 (3.1–21.7)	8.1 (4.5–14.7)	4	0	4782.1	4839.5	8.44 (3.1–22.3)	0
Colorectal cancer	1	11	4910.2	13,539	2.0 (0.3–14.5)	8.1 (4.5–14.7)	1	2	4787.3	4838.6	2.1 (0.3–14.8)	4.1 (1.0–16.5)
Esophagus cancer	0	1	4911.7	13,561.2	0	0.7 (0.1–5.2)	0	1	4788.8	4837.1	0	2.1 (0.3–14.7)
Pancreas cancer	2	4	4911.3	13,561.4	4.1 (1.0–16.3)	2.9 (1.1–7.9)	2	0	4788.3	4839.5	4.2 (1.0–16.7)	0
Lung cancer	0	13	4911.7	13,541.7	0	9.6 (5.6–16.5)	0	2	4788.8	4832.8	4.1 (1.0–16.5)	0
Bladder cancer	1	3	4909.6	13,562.1	2.0 (0.3–14.5)	2.2 (0.7–6.9)	1	0	4786.7	4839.5	2.1 (0.3–14.8)	0
Prostate cancer	1	5	2834.2	8547.2	3.6 (0.5–25.0)	5.8 (2.14–14.1)	1	2	2786.5	2873.2	3.6 (0.5–25.5)	7.0 (1.7–27.8)
Breast cancer	5	7	2069.3	4987.8	24.2 (10.1–58.1)	14.0 (6.7–29.4)	5	2	1994.0	1955.3	25.1 (10.4–30.2)	10.2 (2.6–40.9)
Cervical cancer	0	2	2076.8	5004.3	0	4.0 (1.0–16.0)	0	0	2001.6	1962.3	0	0
Other cancers	14	38	4883.9	13,501.8	28.7 (17.0–48.4)	28.1 (20.5–38.7)	14	6	4761	4829.8	29.4 (17.4–49.7)	12.4 (5.6–27.7)

*SGLT2i* sodium-glucose cotransporter-2 inhibitor, *FLD* fatty liver disease, *T2DM* type 2 diabetes mellitus, *CVH* chronic viral hepatitis, *PS* propensity score, *HR* hazard ratio, *CI* confidence interval.

**Figure 3:**
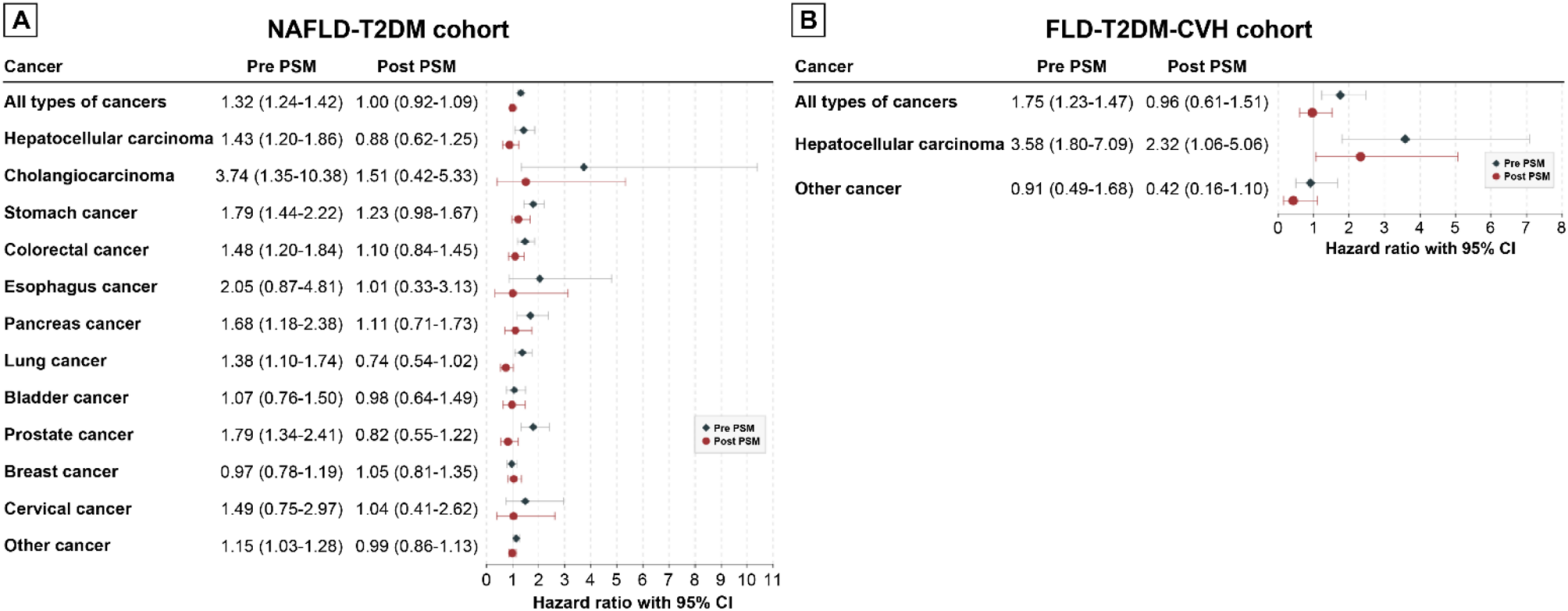
Forrest plots of the hazard ratio of each cancer according to SGLT2i usage in the NAFLD-T2DM cohort and FLD-T2DM-CVH cohort. (**A**) NAFLD-T2DM cohort. (**B**) FLD-T2DM-CVH cohort. *SGLT2i* sodium-glucose cotransporter-2 inhibitor, *PSM* propensity score matching, *NAFLD* non-alcoholic fatty liver disease, *T2DM* type 2 diabetes mellitus, *FLD* fatty liver disease, *CVH* chronic viral hepatitis.

While the IR per 10,000 person-years for HCC increased by more than tenfold in the FLD-T2DM-CVH cohort, the number of cases of other cancer types decreased as the cohort size diminished. As shown in Table 5, for several cancer types, the number of cases was less than 10. Due to concerns such as lack of statistical power, risk of overestimation, adherence to the Events Per Variable rule, and model fit issues, HRs could not be calculated for these types of cancer. We could analyze HR of HCC, “total cancer”, and “other cancer” in both pre- and post-PM matched cohorts. The risk of HCC occurrence in non-SGLT2i users was significantly higher in both cohorts; before matching [crude HR = 3.58 (1.80–7.09)] and in the PS-matched cohort [adjusted HR = 2.32 (1.06–5.06)]. The risk of “total cancer” showed significant HR in the pre-matched cohort; however, this significance disappeared in the post-PS-matched cohort (Fig. 3B).

### Survival analysis of HCC and other cancers in the PS matched FLD-T2DM-CVH cohort according to SGLT2i usage

Figure 2b displays the Kaplan–Meier curves comparing HCC occurrence between SGLT2i and non-SGLT2i users within the PS-matched FLD-T2DM-CVH cohort. SGLT2i users had a significantly lower risk of developing HCC (*P* = 0.03). There were no significant differences in the occurrence of “total cancers” and “other cancers” between the two groups (Fig. S3).

Subsequently, Cox proportional hazards analysis was conducted in the PS-matched FLD-T2DM-CVH cohort (Table 6). In the univariate Cox regression analysis, older age and comorbidities such as dyslipidemia, heart failure, and liver cirrhosis, as well as the use of SGLT2i, statins, and antiviral treatment, were significantly associated with the occurrence of HCC. To adjust for covariates, we performed a multivariate Cox regression analysis by entering variables with a *P* value < 0.1 from the univariate analysis. Sex was also included in the multivariate analysis, although it was not a significant factor in the univariate analysis. This is because it is considered a basic variable for adjustment. In multivariate analysis, SGLT2i usage [HR = 2.22 (1.01–4.87), *P* = 0.047] was identified as an independent risk factor of HCC occurrence along with older age [HR = 1.07 (1.03–1.10), *P* < 0.001], male sex [HR = 2.23 (1.00–5.26), *P* = 0.049], and liver cirrhosis [HR = 7.33 (3.31–16.21), *P* < 0.001]. The C-index of this model was 0.882 with an SE of 0.056.

**Table 6:** Univariate and multivariate Cox regression analysis to identify risk factors associated with HCC occurrence in the PS matched FLD-T2DM-CVH cohort (N = 2798).

Variables	Univariate			Multivariate		
	HR	95% CI	P value	HR	95% CI	P value
Age, year	1.09	1.02–1.09	0.004	1.07	1.03–1.10	< 0.001
Sex, male	1.95	0.87–4.39	0.105	2.30	1.00–5.26	0.049
SGLT2i use, no	2.32	1.06–5.06	0.035	2.22	1.01–4.87	0. 047
CCI, ≤ 2 vs 3	0.84	0.40–1.81	0.671			
Comorbidities						
Hypertension	0.96	0.47–1.96	0.907			
Dyslipidemia	0.41	0.20–0.85	0.017	0.61	0.27–1.36	0.226
Heart failure	3.07	0.93–10.13	0.065			
Cerebrovascular disease	0.78	0.11–5.70	0.803			
Peripheral vascular disease	0.96	0.29–3.16	0.943			
Alcoholic liver disease	2.24	0.68–7.39	0.185			
Liver cirrhosis	12.47	5.93–26.23	< 0.001	7.33	3.31–16.21	< 0.001
Chronic viral hepatitis B	1.77	0.72–4.32	0.213			
Chronic viral hepatitis C	1.17	0.45–3.06	0.746			
Co-medications						
Aspirin	0.83	0.25–2.750	0.766			
Statin	0.29	0.12–0.71	0.006	0.37	0.14–1.05	0.006
Ezetimibe	0.35	0.05–2.53	0.295			
Angiotensin II receptor blocker	0.86	0.41–1.80	0.681			
Calcium channel blocker	1.07	0.47–2.40	0.870			
Beta-blocker	1.12	0.39–3.19	0.840			
Metformin	1.62	0.72–3.65	0.241			
Antiviral treatment	2.80	1.25–6.29	0.013	2.02	0.86–4.76	0.108
Level of antidiabetic treatment, level 1 vs ≥ 2	0.66	0.20–2.18	0.499			

*HCC* hepatocellular carcinoma, *PSM* propensity score matching, *FLD* fatty liver disease, *T2DM* type 2 diabetes mellitus, *CVH* chronic viral hepatitis, *NAFLD* non-alcoholic fatty liver disease, *SGLT2i* sodium-glucose cotransporter-2 inhibitor.

Concordance index = 0.882 (standard error = 0.056).

## Discussion

This study undertook a comprehensive analysis using large-scale healthcare data to investigate the influence of SGLT2i on cancer development, with emphasis on HCC, in a cohort with co-existing FLD and T2DM. By leveraging high-quality data from the HIRA Service of Korea. This study enriches the field with valuable insights into practical implications and outcomes in the clinical setting. Our findings in the NAFLD-T2DM cohort indicated no significant differences in the incidence of HCC and other types of cancers based on SGLT2i use. However, in the HCC high-risk group of patients, the FLD-T2DM-CVH cohort, the use of SGLT2i was significantly associated with a lower incidence of HCC, even after PS matching and multivariate Cox analysis, highlighting its potential protective effect in this particular subgroup.

A previous systematic review and meta-analysis investigating the association between SGLT2i and cancer risk in T2DM patients found no significant increase in the overall cancer risk, consistent with our findings in the NAFLD-T2DM cohort^17^. This prior research, encompassing 46 randomized controlled trials, indicated an increased risk of bladder cancer with SGLT2 inhibitor use but suggested a potential protective effect against gastrointestinal cancers. However, the authors state that further long-term studies are recommended owing to the short-term nature of the trials included in the study. In our study on patients with FLD and T2DM, the use of SGLT2i was not associated with an increased risk of bladder cancer, and the potential protective effect against gastrointestinal cancer was not statistically significant. Chou et al.^20^ reported a protective effect of SGLT2i against HCC compared to dipeptidyl peptidase-4 inhibitors in T2DM patients using data from Hong Kong's National Health Care System. In our study, using data from the Korean HIRA Service, we initially observed a trend towards lower crude IRs of HCC and other cancer types among SGLT2i users within the NAFLD-T2DM cohort. In addition, a significant increase in the HRs of various types of cancers, including HCC, was observed in non-SGLT2i users before matching. However, this trend did not reach statistical significance after PS matching, which was adjusted for discrepancies in person-years attributable to the relatively recent introduction of SGLT2i compared to other oral hypoglycemic agents (OHA). This may be due to differences in the observed person-years between SGLT2i users and non-users. Specifically, SGLT2i users demonstrated relatively shorter person-years than non-users, resulting in an apparent increase in the IR of various cancers in the SGLT2i user group before matching.

While SGLT2i did not demonstrate a statistically significant association with HCC incidence in patients with NAFLD and T2DM, multivariate Cox analysis identified several factors associated with increased HCC risk in this population. These included older age, male sex, presence of hypothyroidism, and liver cirrhosis. Furthermore, the use of statins and fibrates has been associated with a lower incidence of HCC. This observation aligns with the existing research, underscoring the potential protective effects of statins and fibrates against HCC. Previous research has demonstrated that statins may confer a protective benefit in the chemoprevention and treatment of several cancers, including HCC^21–24^. Recently, Zou et al.^25^ suggested an association between statin and reduced risk of HCC development in NAFLD patients by using the Optum de-identified Clinformatics database. Additionally, a large-scale case–control study in Taiwan revealed a significant inverse association between fibrate use and the incidence of liver cancer^26^. The study demonstrated that fibrate use was associated with significantly lower odds of liver cancer in a dose-dependent manner, indicating a protective effect of fibrates against liver cancer. While our study contributes to the understanding of SGLT2i's role in various types of cancer risk, particularly in a specific cohort of patients with NAFLD and T2DM, it also highlights the importance of considering the protective effects of other medications, such as statins and fibrates, in managing HCC risk in this cohort.

In our FLD-T2DM-CVH cohort, we noted a notably higher crude incidence rate of HCC compared to the NAFLD-T2DM cohort. This difference is attributed not only to viral infection but also to variations in HCC screening strategies for both cohorts. CVH is a well-known risk factor for HCC, and it is recommended by various expert groups that patients with CVH should undergo biannual HCC screening^27–29^. On the other hand, in patients without CVH or liver cirrhosis, regular HCC screening is not recommended. Considering the significant differences in HCC risk and HCC screening strategies based on CVH status, we conducted separate analyses for patients with CVH and those NAFLD patients without CVH to minimize potential biases. Interestingly, within the CVH cohort with higher HCC risk, we noted a pronounced protective effect of SGLT2i against development of HCC. This finding is in line with the concepts of risk difference effect and relative risk reduction, suggesting that therapeutic interventions might offer greater absolute benefits in populations at a higher baseline risk^30,31^. The underlying theory suggests that individuals at an elevated risk of a condition may gain more from interventions due to their higher initial risk, potentially preventing a greater number of adverse outcomes^31^. Despite the limitations of our study design and dataset which prevent a detailed statistical analysis to fully quantify this effect, the observed trend highlights the importance of considering baseline risk when evaluating treatment outcomes. This insight is particularly pertinent for clinicians seeking to optimize therapeutic strategies for patients with diverse risk profiles, emphasizing the need for tailored approaches based on individual patient risk factors. Further research is needed to explore this differential effect more comprehensively, possibly by incorporating more detailed data on baseline risk and utilizing statistical methods to assess the interaction effects between treatment efficacy and specific risk factors for HCC in patients. This finding aligns with a territory-wide cohort study conducted in Hong Kong, which reported that SGLT2i use was associated with a lower risk of HCC development in patients with co-existing T2DM and chronic hepatitis B infection^32^. These results suggest the potential protective effects of SGLT2 inhibitors against HCC development in high-risk patients, reinforcing the importance of targeted therapeutic strategies for managing HCC risk in patients with diabetes and chronic viral hepatitis.

The strength of our study lies in its large sample size and utilization of a national database, enabling a robust statistical approach and enhancing the generalizability of our findings. Nevertheless, we acknowledge the presence of inherent limitations, notably the study's retrospective and observational nature, which could introduce biases and the potential for residual confounding factors that might not be fully eliminated through statistical adjustments. In addition to, critical individual patient variables, such as height, weight, and blood glucose levels, which can significantly influence the outcomes, were not directly measured in our study. To mitigate these constraints, we incorporated several variables capable of indirectly representing the baseline health status of patients, including diagnoses related to obesity and the intensity of glycemic control treatments. Notably, in the Korean healthcare system, the prescription of OHAs and insulin is determined by initial HbA1c levels, offering a surrogate marker for assessing patients' baseline glycemic control. This methodology, while not directly measuring each variable, provides a practical and indirect assessment of patients' health conditions that could address, at least partially, some of the limitations mentioned. Furthermore, the potential underdiagnosis of early-stage HCC among non-cirrhotic patients without CVH presents an additional limitation. Our reliance on ICD-10 codes for identifying FLD, T2DM, and any cancers might not capture all instances of early-stage HCC, especially given the lack of established recommendations for HCC screening in non-cirrhotic patients. To address this concern, we employed a wash-out period strategy, however, we recognize that this measure cannot fully overcome the challenges associated with underdiagnosis of early-stage HCC. It indicates the need for future studies to develop more precise diagnostic criteria and screening protocols for this patient population.

In conclusion, within the NAFLD-T2DM cohort, SGLT2i did not demonstrate a statistically significant effect in reducing the risk of developing HCC. In contrast, our analysis within the FLD-T2DM-CVH cohort indicates a significant association between SGLT2i use and a decreased risk of HCC, highlighting their potential as a preventive strategy in patients with a higher risk profile of HCC. Nevertheless, it is important to recognize that our study is based on retrospective cohort data, underscoring the need for future research through prospective cohort studies to further validate these findings.

## Methods

### Data source

We used a dataset from the HIRA database of the Republic of Korea between January 1, 2014, and December 31, 2021. The dataset contained comprehensive information from both inpatient and outpatient medical claims, including details such as prescription drug utilization, diagnostic and treatment codes, and primary and secondary diagnosis codes.

### Study design

This study was designed as a comparative cohort study to evaluate the implications of SGLT2 inhibitor prescription on HCC incidence in patients diagnosed with FLD and T2DM. Figure 1 shows the flowchart of this study. Data were extracted from eligible patients. The eligibility criteria for the study were as follows: (1) patients diagnosed with co-existing FLD and T2DM, and (2) patients receiving treatment with one to three types of OHA. Patients diagnosed with FLD or T2DM were identified based on medical diagnoses according to the International Statistical Classification of Diseases and Related Health Problems, 10th Revision (ICD-10). Individuals who met the following criteria were excluded: those diagnosed with any malignancy or those who underwent liver transplantation before cohort entry or within the year after cohort entry, considering the lag period to eliminate the possibility of detection of already existing cancers. Patients with cohort entry day in 2014 were excluded because they did not meet the criteria for assessing baseline characteristics in the year prior to cohort entry. Patients with a cohort entry day of 2021 were also excluded due to the lack of a minimum one-year follow-up period to evaluate cancer development. Patients who had a history of any OHA or insulin prescription within 1 year before cohort entry were also excluded.

Patients treated with SGLT2i for more than 90 days since cohort entry were categorized into SGLT2i users, while those who never used SGLT2i during 2014–2021 were categorized into the comparative group non-SGLT2i users. The index date was defined as the cohort entry day, which was set as the first date of SGLT2i or other OHA prescriptions. In South Korea, OHA is prescribed according to the insurance coverage criteria of the National Health Insurance Service. The insurance coverage criteria were based on the patient's glycemic control status, as represented by hemoglobin A1c(HbA1c)^33^. Thus, the number of prescribed OHA or insulin use was closely related to the glycemic control status in each patient. Therefore, the use of multiple OHA or insulin suggests that patients with diabetes require more intensive treatment to achieve adequate glycemic control. Furthermore, we classified the patients according to the number of prescribed OHA and insulin usage during the 90 days after cohort entry to reflect the glycemic control level at the time of cohort entry; level 1—one or two OHA had been taken, Level 2—three classes of OHA had been taken without insulin, and level 3—administration of insulin in combination with other OHA. The index year, age at cohort entry, sex, level of antidiabetic treatment 90 days after cohort entry, comorbidities, Charlson Comorbidity Index (CCI), and prescribed drugs during the year prior to cohort entry were analyzed as baseline characteristics.

### Cohort definition

We analyzed two distinct patient cohorts with concurrent FLD and T2DM. The patients presenting with both FLD and T2DM, who also have CVH, are categorized into a higher risk group for HCC, necessitating bi-annual HCC screenings for this population. Conversely, T2DM-NAFLD patients without CVH or liver cirrhosis are not classified as being at high risk for HCC, and thus, regular HCC screenings using ultrasound are not routinely recommended for them. To address the disparities in risk and screening frequencies between patients with CVH and those with only NAFLD, we conducted separate analyses for these groups to mitigate any biases arising from these differences. The first, termed the NAFLD-T2DM cohort, was identified by excluding patients with other causes of chronic liver diseases at baseline, such as CVH, alcoholic liver disease, and autoimmune liver disease including primary biliary cholangitis and autoimmune hepatitis, aligning with the definition of NAFLD. The second cohort, the FLD-T2DM-CVH cohort, included patients diagnosed with CVH in addition to concurrent FLD and T2DM. CVH, alcoholic liver disease, primary biliary cholangitis, and autoimmune hepatitis were diagnosed based on the presence of these diagnoses in medical records during the year prior to cohort entry. Additionally, patients were considered to have received antiviral treatment if they had been prescribed antiviral agents for hepatitis B or C within the year prior to cohort entry. 

### Outcome

The primary outcome of the present study was a diagnosis of any malignancy, which was indicated by the C code in the ICD-10, and registration of catastrophic illness coverage in the national health insurance system for the corresponding malignancies. All eligible patients were followed up from the index date until the occurrence of the primary outcome or the study end date (31st December 2021), whichever occurred first. In this study, we evaluated the occurrence of a spectrum of cancer types: HCC, Cholangiocarcinoma (CCC), and various gastrointestinal cancers (stomach, colorectal, esophageal, and pancreatic), along with lung, bladder, prostate, breast, and cervical cancers. We also included a category termed “other cancers” to encompass less common or unspecified cancer sites. Furthermore, we assessed the combined incidence rate of these malignancies, referred to as “total cancer” incidence, to provide an aggregate measure of cancer diagnoses in our study.

### Statistical analyses

To thoroughly evaluate the baseline characteristics across differing groups in our study, we meticulously applied descriptive statistical techniques. These techniques were used to analyze a wide array of baseline covariates, including age, sex, the intensity of antidiabetic treatment, an array of comorbid conditions, the Charlson Comorbidity Index (CCI), and any co-medication regimes. By employing the absolute standardized mean difference (aSMD) with a threshold set at 0.1 or higher, we successfully pinpointed notable discrepancies between the study groups, ensuring a rigorous comparison basis.

To rigorously adjust for potential confounding factors and balance the comparison groups, we meticulously calculated propensity scores. This was achieved using logistic regression, factoring in critical variables such as age, sex, the index year of study entry, the CCI score, medical histories of hypertension and liver cirrhosis, and the specific level of antidiabetic treatment within the NAFLD-T2DM cohort. Similarly, for the FLD-T2DM-CVH cohort, additional variables including medical histories of hypertension, dyslipidemia, heart failure, coronary artery disease, alcoholic liver disease, chronic hepatitis B, chronic hepatitis C, and the administration history of ACE inhibitors, ARBs, statins, and ezetimibe were considered, alongside the level of antidiabetic treatment. Following this, a precise 1:1 propensity score (PS) matching was executed without replacement using the nearest-neighbor matching algorithm, applying a caliper width of 0.02 to ensure close matches.

Subsequently, we determined the incidence rate (IR) of each cancer type within the study groups, presenting these rates as cases per 10,000 person-years to provide a clear understanding of cancer development risk.

For a comparative analysis of the effect of SGLT2 inhibitors on HCC and other cancer types’ development, Kaplan–Meier curves were plotted, and log-rank tests were utilized, offering a visual and statistical representation of the time-to-event data. To further refine our understanding, both univariate and multivariate Cox proportional hazard regression analyses were conducted. These analyses aimed to estimate hazard ratios [HR] and their 95% confidence intervals [CI] based on baseline variables such as sex, age at cohort entry, detailed comorbidities, and the use of SGLT2i, along with antiplatelet, antihypertensive, and antidyslipidemic agents. The multivariate Cox regression analysis included variables that exhibited a P value of < 0.1 in the univariate analysis, a strategic choice to ensure that all potential predictors of interest showing a trend towards association were considered, even if they did not meet the conventional significance threshold.

These comprehensive statistical analyses were performed using advanced software tools, namely SAS version v9.4 (SAS Institute, Inc., Cary, NC, USA) and R version 4.3.2 (Boston, MA, USA), to ensure the utmost accuracy and reliability of our findings.

### Ethics approval statement

This study was performed according to the Declaration of Helsinki. This retrospective study utilized data from the Health Insurance Review and Assessment Service (HIRA) in South Korea. The institutional review board (IRB) of Ajou university hospital granted an informed consent waiver due to the study's nature and use of de-identified data. Ethical approval was given by the Ajou University IRB, recognizing that patient confidentiality and privacy were upheld, in line with ethical guidelines for retrospective research (AJOUIRB-EX-2023-179).

### Patient consent statement

Patient consent was waived for this study as it exclusively utilized anonymized data, ensuring the privacy and confidentiality of individual participants.

### Supplementary Information

Supplementary Information.

## Data Availability

The data that support the findings of this study are available from the Health Insurance Review and Assessment Service (HIRA) of South Korea but restrictions apply to the availability of these data, which were used under license for the current study, and so are not publicly available. Data are however available from the authors upon reasonable request and with permission of the Health Insurance Review and Assessment Service (HIRA) of South Korea.

## References

[1] Cotter TG, Rinella M (2020). Nonalcoholic fatty liver disease 2020: the state of the disease. Gastroenterology.

[2] Han SK, Baik SK, Kim MY (2023). Non-alcoholic fatty liver disease: Definition and subtypes. Clin. Mol. Hepatol..

[3] Dharmalingam M, Yamasandhi PG (2018). Nonalcoholic fatty liver disease and type 2 diabetes mellitus. Indian J. Endocrinol. Metab..

[4] Cho HJ (2019). Improvement of nonalcoholic fatty liver disease reduces the risk of type 2 diabetes mellitus. Gut Liver.

[5] Kanwal F (2018). Risk of hepatocellular cancer in patients with non-alcoholic fatty liver disease. Gastroenterology.

[6] Nakatsuka T, Tateishi R (2023). Development and prognosis of hepatocellular carcinoma in patients with diabetes. Clin. Mol. Hepatol..

[7] Wang C (2012). Increased risk of hepatocellular carcinoma in patients with diabetes mellitus: A systematic review and meta-analysis of cohort studies. Int. J. Cancer.

[8] Torres MCP (2022). Diabetes medications and risk of HCC. Hepatology.

[9] van Baar MJB (2018). SGLT2 inhibitors in combination therapy: From mechanisms to clinical considerations in type 2 diabetes management. Diabetes Care.

[10] Shao SC, Kuo LT, Chien RN, Hung MJ, Lai EC (2020). SGLT2 inhibitors in patients with type 2 diabetes with non-alcoholic fatty liver diseases: An umbrella review of systematic reviews. BMJ Open Diabetes Res. Care.

[11] Dwinata M, Putera DD, Hasan I, Raharjo M (2020). SGLT2 inhibitors for improving hepatic fibrosis and steatosis in non-alcoholic fatty liver disease complicated with type 2 diabetes mellitus: A systematic review. Clin. Exp. Hepatol..

[12] Arvanitakis K, Koufakis T, Kotsa K, Germanidis G (2022). The effects of sodium-glucose cotransporter 2 inhibitors on hepatocellular carcinoma: From molecular mechanisms to potential clinical implications. Pharmacol. Res..

[13] Komiya C (2016). Ipragliflozin improves hepatic steatosis in obese mice and liver dysfunction in type 2 diabetic patients irrespective of body weight reduction. PLoS One.

[14] Finck BN (2018). Targeting metabolism, insulin resistance, and diabetes to treat nonalcoholic steatohepatitis. Diabetes.

[15] Wong C (2021). Sodium-glucose co-transporter 2 inhibitors for non-alcoholic fatty liver disease in Asian patients with type 2 diabetes: a meta-analysis. Front. Endocrinol. (Lausanne).

[16] Bea S (2023). Sodium-glucose cotransporter-2 inhibitors and risk of hepatocellular carcinoma among patients with type 2 diabetes. Clin. Gastroenterol. Hepatol..

[17] Tang H (2017). SGLT2 inhibitors and risk of cancer in type 2 diabetes: A systematic review and meta-analysis of randomised controlled trials. Diabetologia.

[18] Belle A (2015). Big data analytics in healthcare. Biomed. Res. Int..

[19] Kim JA, Yoon S, Kim LY, Kim DS (2017). Towards actualizing the value potential of Korea Health Insurance Review and Assessment (HIRA) data as a resource for health research: Strengths, limitations, applications, and strategies for optimal use of HIRA data. J. Korean Med. Sci..

[20] Chou OHI (2022). Lower risks of sodium glucose cotransporter 2 (SGLT2) inhibitors compared to dipeptidyl peptidase-4 (DPP4) inhibitors for new-onset non-alcoholic fatty liver disease and hepatocellular carcinoma in type 2 diabetes mellitus: A population-based study. Preprint.

[21] Lonardo A, Loria P (2012). Potential for statins in the chemoprevention and management of hepatocellular carcinoma. J. Gastroenterol. Hepatol..

[22] Demierre MF, Higgins PD, Gruber SB, Hawk E, Lippman SM (2005). Statins and cancer prevention. Nat. Rev. Cancer.

[23] Mansourian PG (2014). Effects of statins on the risk of hepatocellular carcinoma. Gastroenterol. Hepatol. (N. Y.).

[24] Goh MJ, Sinn DH (2022). Statin and aspirin for chemoprevention of hepatocellular carcinoma: Time to use or wait further?. Clin. Mol. Hepatol..

[25] Zou B, Odden MC, Nguyen MH (2023). Statin use and reduced hepatocellular carcinoma risk in patients with nonalcoholic fatty liver disease. Clin. Gastroenterol. Hepatol..

[26] Li IH (2019). Inverse association of fibrates and liver cancer: A population-based case-control study in Taiwan. J. Clin. Pharmacol..

[27] Cho Y, Kim BH, Park JW (2023). Overview of Asian clinical practice guidelines for the management of hepatocellular carcinoma: An Asian perspective comparison. Clin. Mol. Hepatol..

[28] Singal AG (2023). AASLD Practice Guidance on prevention, diagnosis, and treatment of hepatocellular carcinoma. Hepatology.

[29] Kinsey E, Lee HM (2024). Management of hepatocellular carcinoma in 2024: The multidisciplinary paradigm in an evolving treatment landscape. Cancers (Basel).

[30] Schechtman E (2002). Odds ratio, relative risk, absolute risk reduction, and the number needed to treat—Which of these should we use?. Value Health.

[31] Austin PC (2010). Absolute risk reductions, relative risks, relative risk reductions, and numbers needed to treat can be obtained from a logistic regression model. J. Clin. Epidemiol..

[32] Lee CH (2023). SGLT2i reduces risk of developing HCC in patients with co-existing type 2 diabetes and hepatitis B infection: A territory-wide cohort study in Hong Kong. Hepatology.

[33] Choi JH (2023). 2023 clinical practice guidelines for diabetes mellitus of the Korean Diabetes Association. Diabetes Metab. J..

