# Supplementary material for: SGLT2i impact on HCC incidence in patients with fatty liver disease and diabetes: a nation-wide cohort study in South Korea
Source: Sci Rep. 2024 Apr 29;14:9761. doi: 10.1038/s41598-024-60133-3 (PMC11058854; doi:10.1038/s41598-024-60133-3)
Supplement: Supplementary file 1 — Supplementary Information. [file 41598_2024_60133_MOESM1_ESM.docx]

**Supplementary materials**

**Supplementary Figure S1**. Love Plot of Covariate Balance Post-Propensity Score Matching


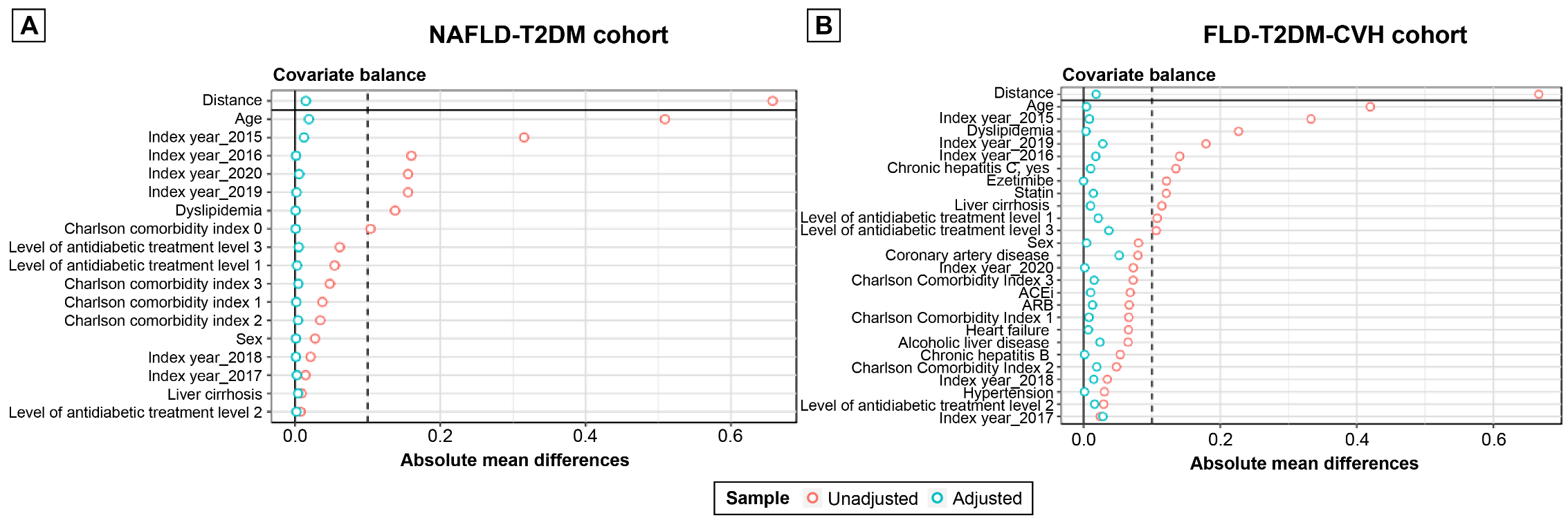


**Supplementary Figure S2**. Comparison of Kaplan-Meier curves of each cancer risk other than hepatocellular carcinoma according to SGLT2i exposure in PS-matched NAFLD-T2DM cohort


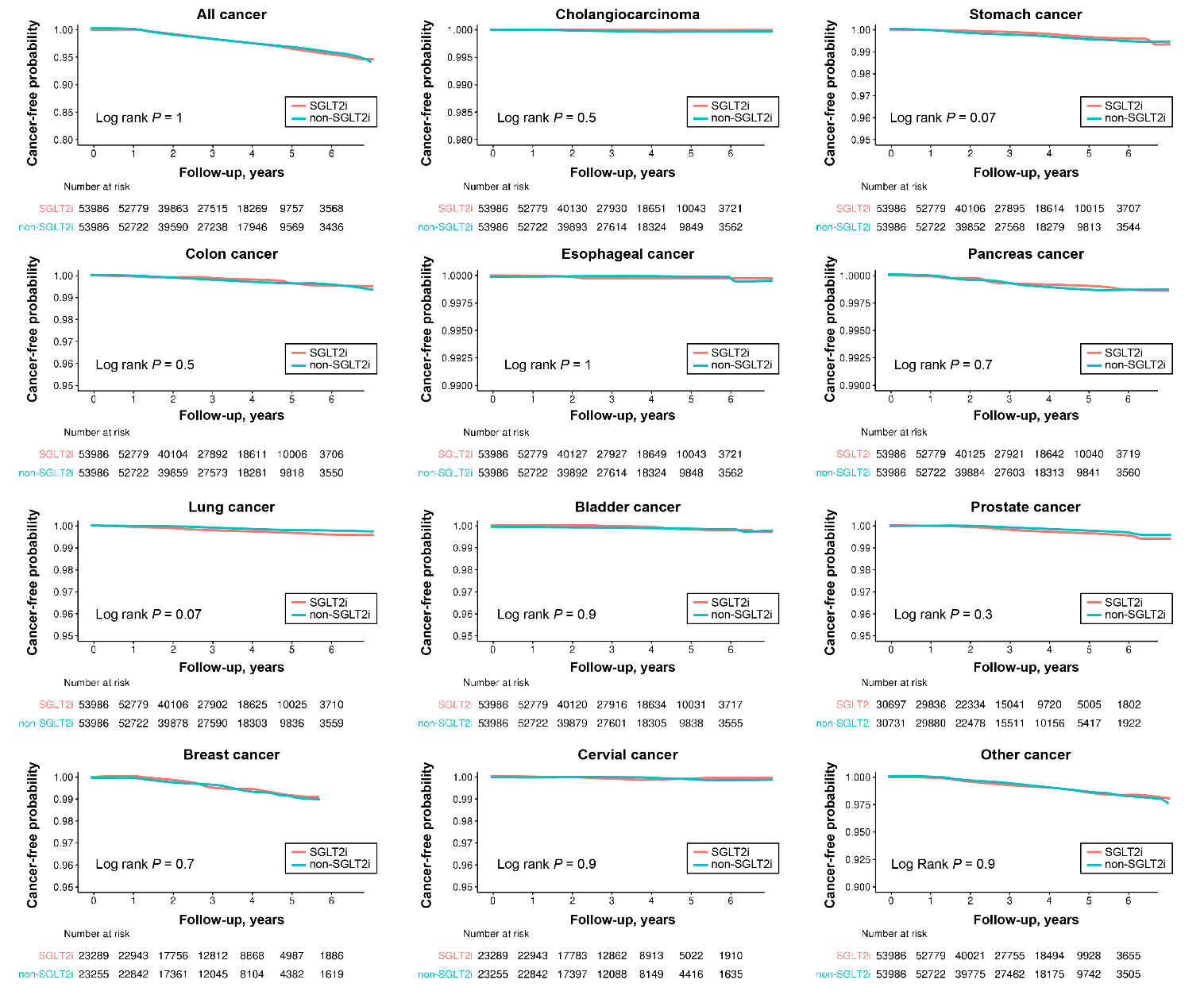


**Supplementary Figure S3.** Comparison of Kaplan-Meier curves of all cancer and other type of cancer risk other than hepatocellular carcinoma according to SGLT2i exposure in PSM adjusted FLD-T2DM-CVH cohort


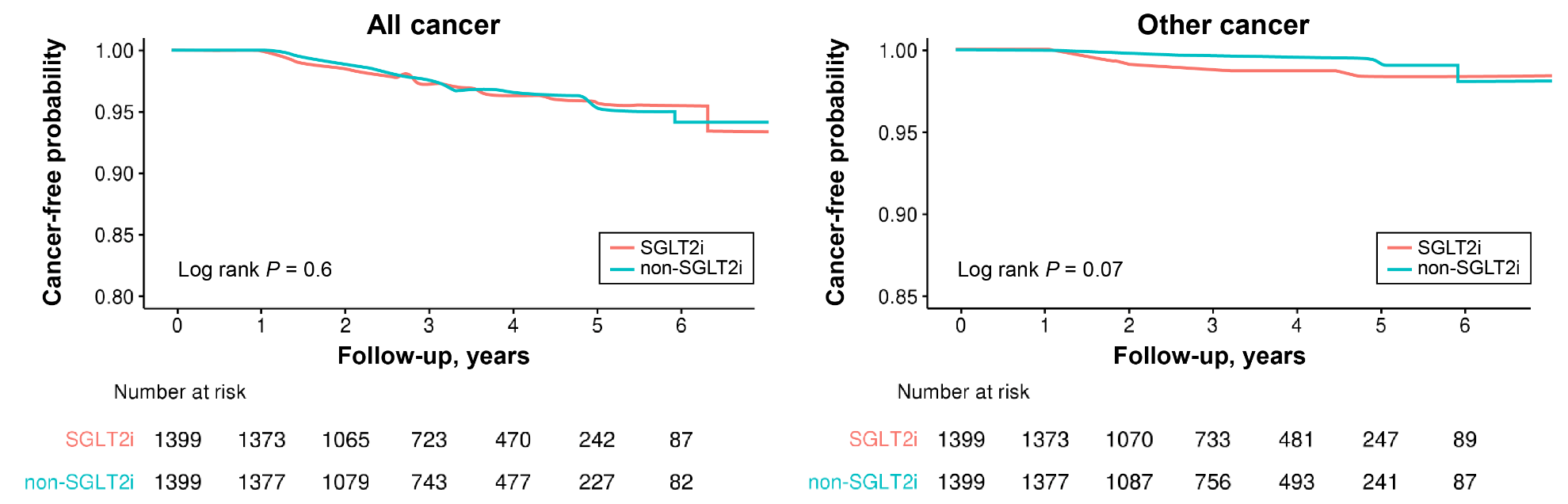


**Supplementary Table S1.** The resulting balance table after propensity score matching in the NAFLD-T2DM cohort

|  | Type | Mean Pre-Match Treatment Group | Standard Deviation Pre-Match Treatment Group | Mean Pre-Match Control Group | Standard Deviation Pre-Match Control Group | Unadjusted Difference | Mean Post-Match Treatment Group | Standard Deviation Post-Match Treatment Group | Mean Post-Match Control Group | Standard Deviation Post-Match Control Group | Adjusted Difference |
| --- | --- | --- | --- | --- | --- | --- | --- | --- | --- | --- | --- |
| Distance | Distance | 0.249919 | 0.127233 | 0.34676 | 0.147294 | 0.657467 | 0.334986 | 0.138231 | 0.337195 | 0.139294 | 0.015002 |
| Age | Contin. | 56.29221 | 12.01754 | 49.71535 | 12.91496 | 0.509244 | 50.61223 | 12.47766 | 50.36504 | 12.54688 | 0.01914 |
| Sex | Binary | 0.582197 | NA | 0.56846 | NA | 0.027735 | 0.56924 | NA | 0.56861 | NA | 0.001272 |
| Index year_2015 | Binary | 0.157479 | NA | 0.074592 | NA | 0.315477 | 0.073667 | NA | 0.076927 | NA | 0.012408 |
| Index year_2016 | Binary | 0.172152 | NA | 0.120082 | NA | 0.160185 | 0.122717 | NA | 0.123162 | NA | 0.001368 |
| Index year_2017 | Binary | 0.164785 | NA | 0.159405 | NA | 0.014697 | 0.162023 | NA | 0.161134 | NA | 0.002429 |
| Index year_2018 | Binary | 0.16604 | NA | 0.174216 | NA | 0.021554 | 0.174823 | NA | 0.175286 | NA | 0.001221 |
| Index year_2019 | Binary | 0.168448 | NA | 0.234373 | NA | 0.155627 | 0.23093 | NA | 0.23006 | NA | 0.002055 |
| Index year_2020 | Binary | 0.171096 | NA | 0.237332 | NA | 0.155684 | 0.235839 | NA | 0.233431 | NA | 0.00566 |
| Level of antibiatetic treatement, level 1 | Binary | 0.873803 | NA | 0.854545 | NA | 0.054622 | 0.858223 | NA | 0.857222 | NA | 0.002837 |
| Level of antibiatetic treatement, level 2 | Binary | 0.055621 | NA | 0.057432 | NA | 0.007784 | 0.057811 | NA | 0.057348 | NA | 0.00199 |
| Level of antibiatetic treatement, level 3 | Binary | 0.070576 | NA | 0.088022 | NA | 0.061576 | 0.083966 | NA | 0.08543 | NA | 0.005165 |
| Dyslipidmia | Binary | 0.570219 | NA | 0.636471 | NA | 0.137732 | 0.632775 | NA | 0.633127 | NA | 0.000732 |
| Liver cirrhosis | Binary | 0.002778 | NA | 0.002349 | NA | 0.00887 | 0.002593 | NA | 0.00239 | NA | 0.004209 |
| Charlson Comorbidity Index 0 | Binary | 0.090799 | NA | 0.065107 | NA | 0.104138 | 0.066721 | NA | 0.066925 | NA | 0.000826 |
| Charlson Comorbidity Index 1 | Binary | 0.182257 | NA | 0.168119 | NA | 0.037805 | 0.170174 | NA | 0.169507 | NA | 0.001783 |
| Charlson Comorbidity Index 2 | Binary | 0.27731 | NA | 0.29315 | NA | 0.034799 | 0.293706 | NA | 0.291835 | NA | 0.00411 |
| Charlson Comorbidity Index 3 | Binary | 0.449634 | NA | 0.473624 | NA | 0.048047 | 0.469399 | NA | 0.471733 | NA | 0.004674 |

**Supplementary Table S2.** The resulting balance table after propensity score matching in the FLD-T2DM-CVH cohort

|  | Type | Mean Pre-Match Treatment Group | Standard Deviation Pre-Match Treatment Group | Mean Pre-Match Control Group | Standard Deviation Pre-Match Control Group | Unadjusted Difference | Mean Post-Match Treatment Group | Standard Deviation Post-Match Treatment Group | Mean Post-Match Control Group | Standard Deviation Post-Match Control Group | Adjusted Difference |
| --- | --- | --- | --- | --- | --- | --- | --- | --- | --- | --- | --- |
| Distance | Distance | 0.264126 | 0.130171 | 0.358761 | 0.142017 | 0.666362 | 0.347203 | 0.131776 | 0.349789 | 0.133584 | 0.018209 |
| Age | Contin. | 56.45195 | 10.81603 | 51.75694 | 11.18773 | 0.419656 | 52.3138 | 10.98677 | 52.26662 | 10.85315 | 0.004217 |
| Sex | Binary | 0.628432 | NA | 0.588889 | NA | 0.08034 | 0.591851 | NA | 0.593996 | NA | 0.004357 |
| Index year_2015 | Binary | 0.153032 | NA | 0.06875 | NA | 0.332977 | 0.072194 | NA | 0.07005 | NA | 0.008472 |
| Index year_2016 | Binary | 0.165046 | NA | 0.119444 | NA | 0.140561 | 0.117227 | NA | 0.122945 | NA | 0.017626 |
| Index year_2017 | Binary | 0.165046 | NA | 0.174306 | NA | 0.0244 | 0.184417 | NA | 0.173695 | NA | 0.028253 |
| Index year_2018 | Binary | 0.166476 | NA | 0.179861 | NA | 0.034839 | 0.185132 | NA | 0.179414 | NA | 0.014884 |
| Index year_2019 | Binary | 0.174199 | NA | 0.252083 | NA | 0.179308 | 0.234453 | NA | 0.246605 | NA | 0.027976 |
| Index year_2020 | Binary | 0.176201 | NA | 0.205556 | NA | 0.072614 | 0.206576 | NA | 0.207291 | NA | 0.001768 |
| Charlson Comorbidity Index 1 | Binary | 0.046053 | NA | 0.034028 | NA | 0.066302 | 0.03574 | NA | 0.03431 | NA | 0.007882 |
| Charlson Comorbidity Index 2 | Binary | 0.297197 | NA | 0.275694 | NA | 0.048102 | 0.271623 | NA | 0.2802 | NA | 0.019188 |
| Charlson Comorbidity Index 3 | Binary | 0.656751 | NA | 0.690278 | NA | 0.072485 | 0.692638 | NA | 0.68549 | NA | 0.015454 |
| Hypertension | Binary | 0.525744 | NA | 0.540972 | NA | 0.030549 | 0.54253 | NA | 0.541816 | NA | 0.001434 |
| Dyslipidemia | Binary | 0.701945 | NA | 0.79375 | NA | 0.226817 | 0.790565 | NA | 0.789135 | NA | 0.003532 |
| Heart failure | Binary | 0.030892 | NA | 0.044444 | NA | 0.065738 | 0.041458 | NA | 0.042888 | NA | 0.006935 |
| Coronary artery diease | Binary | 0.028032 | NA | 0.044444 | NA | 0.079613 | 0.033595 | NA | 0.044317 | NA | 0.05201 |
| Alcoholic liver disease | Binary | 0.058638 | NA | 0.045139 | NA | 0.065001 | 0.040029 | NA | 0.045032 | NA | 0.024093 |
| Chronic viral hepatitis B | Binary | 0.069794 | NA | 0.045833 | NA | 0.114537 | 0.045032 | NA | 0.047177 | NA | 0.010251 |
| Chhoic viral hepatitis C | Binary | 0.682494 | NA | 0.706944 | NA | 0.053699 | 0.708363 | NA | 0.709078 | NA | 0.00157 |
| Liver cirrhosis | Binary | 0.182494 | NA | 0.136111 | NA | 0.135218 | 0.133667 | NA | 0.137241 | NA | 0.010419 |
| Level of anti-diabetic treatment , level 1 | Binary | 0.881007 | NA | 0.841667 | NA | 0.107728 | 0.852752 | NA | 0.844889 | NA | 0.021531 |
| Level of anti-diabetic treatment , level 2 | Binary | 0.044908 | NA | 0.051389 | NA | 0.029341 | 0.05361 | NA | 0.050036 | NA | 0.016182 |
| Level of anti-diabetic treatment , level 3 | Binary | 0.074085 | NA | 0.106944 | NA | 0.106291 | 0.093638 | NA | 0.105075 | NA | 0.036994 |
| ACEi | Binary | 0.010011 | NA | 0.019444 | NA | 0.068291 | 0.01644 | NA | 0.01787 | NA | 0.01035 |
| ARB | Binary | 0.369279 | NA | 0.402083 | NA | 0.066881 | 0.410293 | NA | 0.40386 | NA | 0.013116 |
| Statin | Binary | 0.412471 | NA | 0.472917 | NA | 0.121026 | 0.468192 | NA | 0.47534 | NA | 0.014312 |
| Ezetimibe | Binary | 0.074085 | NA | 0.1125 | NA | 0.121533 | 0.111508 | NA | 0.111508 | NA | 0 |
